# Characterization of Nutritional Composition, Antioxidative Capacity, and Sensory Attributes of *Seomae* Mugwort, a Native Korean Variety of *Artemisia argyi* H. Lév. & Vaniot

**DOI:** 10.1155/2015/916346

**Published:** 2015-10-13

**Authors:** Jae Kyeom Kim, Eui-Cheol Shin, Ho-Jeong Lim, Soo Jung Choi, Cho Rong Kim, Soo Hwan Suh, Chang-Ju Kim, Gwi Gun Park, Cheung-Seog Park, Hye Kyung Kim, Jong Hun Choi, Sang-Wook Song, Dong-Hoon Shin

**Affiliations:** ^1^Department of Food Science and Nutrition, University of Minnesota, Saint Paul, MN 55108, USA; ^2^Department of Food Science, Gyeongnam National University of Science and Technology, Jinju 660-758, Republic of Korea; ^3^Functional Food Research Center, Korea University, Seoul 136-701, Republic of Korea; ^4^Department of Food and Biotechnology, Korea University, Seoul 136-701, Republic of Korea; ^5^National Institute of Food and Drug Safety Evaluation, Ministry of Food and Drug Safety, Osong 363-700, Republic of Korea; ^6^Department of Physiology, Kyung Hee University, Seoul 130-701, Republic of Korea; ^7^Department of Food Science and Biotechnology, Gachon University, Seongnam 461-701, Republic of Korea; ^8^Department of Microbiology, Kyung Hee University, Seoul 130-701, Republic of Korea; ^9^Department of Food Biotechnology, Hanseo University, Seosan 356-706, Republic of Korea; ^10^Natural Way Company, Pocheon 83-135, Republic of Korea; ^11^Department of Family Medicine, The Catholic University of Korea, Suwon 442-723, Republic of Korea

## Abstract

Few studies have investigated *Seomae* mugwort (a Korean native mugwort variety of *Artemisia argyi* H. Lév. & Vaniot), exclusively cultivated in the southern Korean peninsula, and the possibility of its use as a food resource. In the present study, we compared the nutritional and chemical properties as well as sensory attributes of *Seomae* mugwort and the commonly consumed species *Artemisia princeps* Pamp. In comparison with *A. princeps, Seomae* mugwort had higher contents of polyunsaturated fatty acids, total phenolic compounds, vitamin C, and essential amino acids. In addition, *Seomae* mugwort had better radical scavenging activity and more diverse volatile compounds than *A. princeps* as well as favorable sensory attributes when consumed as tea. Given that scant information is available regarding the *Seomae* mugwort and its biological, chemical, and sensory characteristics, the results herein may provide important characterization data for further industrial and research applications of this mugwort variety.

## 1. Introduction

*Mugworts (the genus Artemisia)* have been widely used as tea, spices, and food ingredients in East Asia. Much attention has been recently paid to their multiple health benefits including anti-tumor-promoting effects [1], induction of apoptosis in various types of cancer cells [2, 3], antidiabetic effects [4], anti-inflammatory effects [5], and anticoagulant/antiplatelet activities [6]. Amongst a plethora of* Artemisia* species,* Artemisia princeps* Pamp., which is widely consumed in Korea, and its bioactive compounds (e.g., eupatilin and jaceosidin) have been most extensively studied in various experimental models [5, 6], yet little information is available regarding the Korean native mugwort variety (also known as* Seomae* mugwort) of* Artemisia argyi* H. Lév. & Vaniot, cultivated in the southern Korean peninsula.

Considering that (1) environmental factors play a significant role in growth as well as the content of active compounds of* Artemisia* species [7], (2) diverse* Artemisia* species have been demonstrated to have varying biological effects [4], and (3) scant information is available regarding the native variety of* A. argyi* (exclusively cultivated in Namhae County, Republic of Korea) and its biological, chemical, and sensory characteristics, it would be important and timely to report the chemical composition and functionality of this variety and the possibility of its use as a food ingredient. Specifically, in the present study both fatty acids and amino acids profiles were analyzed in order to compare contents of essential fatty acids (e.g., linoleic acid) and essential amino acids. Further, antioxidative capacity, vitamin C contents (i.e., a major vitamin of mugworts), and total phenolic compounds were assessed to address potential health promoting effects thereof. In addition, mugwort teas were prepared using* Seomae* mugwort and* A. princeps* and their sensory attributes were analyzed to test potential for the practical use of* mugwort tea*. All parameters of* Seomae* mugwort analyzed in the study were compared with those of* A. princeps*.

## 2. Materials and Methods

### 2.1. Materials

The* Seomae* mugwort (a Korean native variety of* A. argyi*) was kindly provided by the Namhae Agricultural Association Corporation (Namhae, Republic of Korea) where all* Seomae* mugworts harvested in the entire Namhae County are collected. This variety was specifically cultivated in Namhae County, Republic of Korea.* A. princeps* was purchased from a local store (Jinju, Republic of Korea). After being obtained, both mugworts were identified and specimen vouchers were issued by the Department of Agriculture and Herbal Resources of the Gyeongnam National University of Science and Technology (GFA-006 and GFA-007 for* A. princeps* and* Seomae* mugwort, resp.). Leaf samples were completely dried at room temperature, ground, and then stored at −80°C until being analyzed. Heptadecanoic acid (98% purity) and a lipid standard mixture (37 fatty acid methyl esters (FAME)) were from Sigma-Aldrich Co. (St. Louis, MO, USA). Other chemicals were of analytical grade.

### 2.2. Analysis of Free Amino Acids

Briefly, 1 g of each sample was added to 20 mL of pure ethanol and agitated for 10 min. After agitation, samples were centrifuged at 3000 ×g for 20 min and the supernatants were evaporated using a rotary evaporator (R-III; BÜCHI, Postfach, Switzerland). The residues were dissolved in 25 mL of Lithium Loading Buffer (Biochrom Ltd., Cambridge, UK) and incubated for 1 h at 4°C after addition of 20 mg of sulfosalicylic acid. Subsequently, samples were centrifuged at 3000 ×g for 20 min and filtered through a 0.2 *μ*m membrane filter. Samples were analyzed using an amino acid analyzer (L-8900; Hitachi High Tech, Tokyo, Japan) equipped with an ion exchange column (2622PF, 4.6 mm × 60 mm; Hitachi High Tech). The column temperature ranged between 30°C and 70°C and the detection wavelengths were 570 nm and 440 nm.

### 2.3. Fatty Acid Composition

#### 2.3.1. Lipid Extraction

Total lipids were extracted as reported elsewhere with slight modifications [8]. Ten grams of ground samples was suspended in 20 mL of deionized water, 50 mL of methanol, and 25 mL of chloroform, and ~10 mg of hydroquinone was subsequently added. The contents were agitated on an orbital shaker for 2 min at 3000 ×g and the resulting slurry was filtered using a filter paper (Whatman No. 1; GE Healthcare, Little Chalfont, UK). One gram of sodium chloride was added to the filtrate to facilitate phase separation and the filtrate was placed at room temperature overnight. The resulting chloroform phase was then evaporated and samples were stored under a nitrogen headspace at −80°C until being further analyzed.

#### 2.3.2. Fatty Acid Methylation

To analyze fatty acid profiles, FAME were prepared as described previously [9]. In brief, extracted lipids (25 mg) were transferred into a Reacti-Vial (Thermo Fisher Scientific, Rockford, IL, USA) and their mass was accurately measured. The internal standard (heptadecanoic acid in hexane, 1 mg/mL) was added and samples were mixed with 0.5 N sodium hydroxide in methanol followed by flushing with nitrogen gas. The mixtures were then placed in a heating block set at 100°C for 5 min. After cooling, 2 mL of 14% boron trifluoride solution (in methanol) was added to each vial equipped with a Reacti-Vial magnetic stirrer. The vials were vortexed and placed in the Reacti-Block B-1 aluminum block within a Reacti-Therm III Heating/Stirring Module (Thermo Fisher Scientific) at 100°C for 30 min. After derivatization, each sample was extracted with 1.5 mL of hexane.

#### 2.3.3. GC Analysis

An Agilent Technologies (Santa Clara, CA, USA) 7890A Network GC system equipped with a flame ionization detector (FID) was used to quantify fatty acids. Chromatography was performed on an SP-2560 capillary column (100 m × 0.25 mm* i*.*d*., 0.25 *μ*m film thickness; Sigma-Aldrich Co.). The analyses were performed in the constant flow mode. A split liner with glass wool was installed in the injector and the injector temperature was set at 220°C for injection. The FID temperature was set at 240°C, and ultrahigh purity hydrogen (flow rate: 40 mL/min) and scientific-grade air (flow rate: 450 mL/min) were used as the FID fuel gases. The temperature of the oven was initially held at 140°C for 5 min and then was ramped up at 4°C/min to 230°C and maintained at 230°C for an additional 35 min. Triplicate readings were taken.

#### 2.3.4. Fatty Acid Identification

Using the internal standard (heptadecanoic acid), the relative response factor for each FAME was calculated by using the following equation:

(1) $$\begin{array}{c} R_{i}=\frac{\left(Ps_{i}\times Ws_{\text{C}17:0}\right)}{\left(Ps_{\text{C}17:0}\times Ws_{is}\right)}, \end{array}$$

where *R*_*i*_ is the relative response factor for fatty acid *i*, *Ps*_*i*_ is the peak area of individual FAME *i* in the FAME standard solution, *Ws*_C17:0_ is the mass (mg) of heptadecanoic acid FAME in the injected FAME standard solution, *Ps*_C17:0_ is the peak area of heptadecanoic acid FAME in the FAME standard solution, and *Ws*_*is*_ is the mass (mg) of individual FAME *i* in the injected FAME standard solution.

#### 2.3.5. Method Validation for Fatty Acid Analysis

The relative repeatability standard deviation and % relative standard deviation were determined to validate the method for fatty acid analysis in lipid extracts of* Seomae* mugwort and* A. princeps* by assaying Standard Reference Material (SRM) 1849a (Infant/Adult Nutritional Formula) purchased from the National Institute of Standards and Technology (Gaithersburg, MD, USA).

### 2.4. Determination of Total Phenolic Compounds

The total phenolic contents of* Seomae* mugwort and* A. princeps* were compared using a previously described spectrophotometric method with slight modifications [10]. The dried samples were prepared at a concentration of 1 mg/mL of water and then a 40 *μ*L aliquot of each sample was diluted with 200 *μ*L of distilled water. Folin-Ciocalteu's reagent (200 *μ*L; Sigma-Aldrich Co.) was added to the mixture, followed by the addition of 600 *μ*L of sodium carbonate solution (30%, w/v) and 160 *μ*L of distilled water. The mixture was thoroughly mixed and kept in the dark for 2 h at 25°C, after which the absorbance was read at 750 nm. The total phenolic compounds in each sample were determined from interpolation of the calibration curve constructed by using gallic acid solution (0–500 *μ*g/mL).

### 2.5. Analysis of Vitamin C Contents

One gram of ground mugwort sample was added to 1 mL of 10% formic acid and then diluted with 19 mL of 5% formic acid. Samples were thoroughly vortexed and placed at room temperature for 20 min followed by centrifugation (1,000 ×g; 10 min). Resulting supernatants were filtered through a HPLC membrane filter (Sigma-Aldrich Co., Nylon 66 Filter Membranes, 0.45 *μ*m) and injected to a HPLC system (10 *μ*L injection; Shimadzu, Kyoto, Japan). Isocratic method (0.05 M of phosphate buffer and acetonitrile, 60 : 40) was used for the separation of vitamin C using the Bondapak C18 column (Waters, Milford, MA, USA) which was utilized for separation and analytes were monitored at 245 nm wavelength. The standard curve was constructed using the authentic standard for quantification of vitamin C. The *r*-squared value of the standard curve was greater than 0.99.

### 2.6. Antioxidative Capacity Measurement

The antioxidant capacity of* Seomae* mugwort and* A. princeps* was compared using a typical 2,2-diphenyl-1-picrylhydrazyl (DPPH) assay as described elsewhere [11]. In brief, serial dilutions of samples were prepared (200, 400, 600, and 800 *μ*g/mL) and then 80 *μ*L of each sample was added to 320 *μ*L of DPPH solution (0.2 mM, dissolved in pure ethanol). The reactions were performed in an incubator at 37°C and the absorbance was measured at 517 nm. The IC_50_ of each sample was calculated by using the following equation:

(2) Radical scavenging activity (%)=1−the absorbance of the treated samplethe absorbance of control sample×100.

### 2.7. Analysis of Volatile Compound Composition

A Likens and Nickerson–type simultaneous steam distillation and extraction apparatus (SDE) was used for the extraction of volatile compounds according to the method reported elsewhere [12]. Ground samples (100 g) were mixed with distilled water (1 L) followed by the addition of internal standard (1 mL of *n*-pentadecane, 1 mg/mL, Sigma-Aldrich Co.). Atmospheric steam distillation was performed to collect sample volatiles in a 100 mL mixture of *n*-pentane and diethyl ether (1 : 1, v/v) over 3 h at 110°C. Anhydrous sodium sulfate (10 g) was added to the extracts, which were then placed at 4°C overnight. Samples were then filtered and reduced to a volume of 1 mL using a nitrogen evaporator. Concentrated samples were analyzed using a GC fitted with a mass spectrometer (Agilent 7890A and 5975C, resp.), which was operated in electron impact ionization mode (70 eV), scanning a mass range (*m/z*) from 30 to 550 amu. An HP-5MS column (30 m × 0.25 mm,* i.d.* × 0.25 *μ*m film thickness, Agilent Technologies) was used for the analysis. The temperature of the column was maintained at 4°C for the first 5 min and then increased to 200°C at a rate of 5°C/min. The analysis was carried out in the splitless mode, using helium as the carrier gas (1 mL/min flow rate). The injector temperature was 220°C. Separated peaks in the total ionization chromatogram were identified using a database (The NIST 12 Mass Spectrum Library; Gaithersburg, MD, USA) and then confirmed by matching the retention indices (RI) with data from published literature. RI were calculated according to the following formula [13] and based on a series of* n*-alkanes (C8–C20):

(3) $$\begin{array}{c} RI_{x}=100n+100\left(\;\frac{t_{Rx}-t_{Rn}}{t_{Rn+1}-t_{Rn}}\;\right), \end{array}$$

where RI_*x*_ is RI of the unknown compound, *t*_*Rx*_ is retention time of the unknown compound, *t*_*Rn*_ is retention time of the *n*-alkane, and *t*_*Rn*+1_ is retention time of the next *n*-alkane. Each *t*_*Rx*_ is between *t*_*Rn*_ and *t*_*Rn*+1_ (*n* = number of carbon atoms).

### 2.8. Olfactometry Analysis

Separated volatile compounds were further analyzed through an olfactory detection port with a heated mixing chamber (ODP 3; Gerstel, Linthicum, MD, USA). In advance of performing experiments, panels were trained with the instrument operation and data collection. Specifically, they were asked to respond to their perceived intensity of odor through the detection port using a signal generator. The intensity scales of the signal generator ranged from 0 (no perception) to 5 (the strongest perception). To take into account individual variations, 3 trained panels performed an identical experiment and recorded the intensity of each volatile compound isolated from the samples.

### 2.9. Evaluation of Sensory Attributes of* Seomae* Mugwort and* A. princeps*

#### 2.9.1. Study Participants

A total of 15 participants evaluated the sensory attributes of the two* Artemisia* species. All subjects were recruited from the Gyeongnam National University of Science and Technology through fliers and received a gift card incentive for participation. People who discovered themselves having allergy to either* Seomae* mugwort or* A. princeps* were screened prior to the sensory evaluation. The study was approved by the University Institutional Review Board and consent forms were provided to participants. The* demographic* characteristics* are summarized in* Table 1.

#### 2.9.2. Tea Preparation and Sensory Evaluation

To prepare mugwort tea, 5 g of a dried sample was added to 1 L of boiling water and brewed for 5 min. All preparation steps were performed by a professional cook and samples were prepared about 10 min before sensory evaluation. Teas prepared from both species (100 mL each) were provided to each subject. Participants evaluated the teas for perceived color acceptability, flavor acceptability, saltiness, bitterness, sourness, astringency, sweetness, and overall preference using labeled affective magnitude (LAM) scales; the scales were labeled with the phrases “greatest imaginable like,” “like extremely,” “like very much,” “like moderately,” “like,” “neither like nor dislike,” “dislike moderately,” “dislike very much,” “dislike extremely,” and “greatest imaginable dislike.” The scales ranged from 0 (greatest imaginable dislike) to 15 (greatest imaginable like) [14].

### 2.10. Statistical Analysis

All results were expressed as the mean ± standard deviation (SD). The statistical significance between groups (i.e.,* Seomae* mugwort versus* A. princeps*) was tested via Student's *t*-test, using the Statistical Analysis System (SAS; Cary, NC, USA). A *P* value less than 0.05 was considered to be statistically significant.

## 3. Results and Discussion

To compare general nutritional compositions of* Seomae* mugwort and* A. princeps*, we analyzed the content of free amino acids, fatty acids, vitamin C, and total phenolic compounds. First, we found that the content of free amino acids of* A. princeps* was significantly different from that of* Seomae* mugwort (Table 2). Specifically, the content of the essential amino acids valine and phenylalanine was significantly higher in* Seomae* mugwort (by approximately 63% and 41%, resp.) than in* A. princeps*. The content of total essential amino acids was approximately 57% in* A. princeps* and 61% in* Seomae* mugwort. Notably, it has been reported that *γ*-aminobutyric acid (GABA), a nonprotein amino acid, is beneficial for treatment of general anxiety and anxiety disorders [15, 16]. We found that* Seomae* mugwort had approximately 3.8-fold higher content of GABA than* A. princeps*, indicating potential benefits of this variety in medicinal psychopharmacology, which warrants further investigations.

The fatty acid analysis method was validated before determination of the fatty acid composition of* Seomae* mugwort and* A. princeps* (Table 3). The accuracy of the method was calculated based on the percentage of the certified fatty acid content in SRM 1849a and expressed as the percentage of the accepted value. The accuracy ranged from 94.12 to 108.33%, while the reproducibility of the method, indicated by the relative standard deviation (RSD), was higher than 90% for all fatty acids. The complete fatty acid profiles of* Seomae* mugwort and* A. princeps* are shown in Table 4. In total, nine fatty acids, ranging from C16 to C24, were detected based on retention mapping with external standards. These fatty acids were quantified relative to the internal standard (heptadecanoic acid). In* A. princeps*, C18:1 and C18:2 were the most prevalent fatty acids (34.91% and 27.56%, resp.), followed by C18:3 *ω*-6 (9.83%), C16:0 (8.73%), and other fatty acids. Interestingly, the content of C18:3 *ω*-6 was much higher in* Seomae* mugwort (36.36%, Table 4).* Artemisia princeps* had a lower total content of saturated fatty acids than* Seomae* mugwort (27.47% versus 40.79%), while the content of polyunsaturated fatty acids was higher in* Seomae* mugwort, likely due to C18:3 *ω*-6 (Table 4).

The amount of phenolic compounds in* A. princeps* was 49.12 ± 1.23 mg per 100 g of dried material whilst it was much higher (by approximately 50%) in* Seomae* mugwort (74.53 ± 2.08 mg per 100 g, Table 5). Further, the vitamin C content of* Seomae* mugwort was 2-fold higher than that in* A. princeps*. Specifically, it was found that* Seomae* mugwort contains 209.1 ± 3.2 mg of vitamin C per 100 g of dried sample materials (Table 5). We compared the antioxidative capacities of the two mugwort species using the DPPH radical scavenging assay and found that the IC_50_ value of* Seomae* mugwort was 0.55 ± 0.09 mg, whereas* A. princeps* extract required a higher concentration, 0.82 ± 0.12 mg, which is expected given the significantly higher amounts of vitamin C/total phenolic compounds in the* Seomae* mugwort. Generally, the antioxidant activity is closely correlated with the amount of phenolic compounds [17, 18]; this trend was also observed in the present study (Table 5). However, due to the inherent limitations of the method (e.g., nonspecific oxidation by Folin-Ciocalteu's reagent), identification of specific phenolic constituents was not possible in our experiments. Their identification in the future might further elucidate the health benefits of these mugwort species.

Using SDE, 43 volatile compounds were identified in* A. princeps* and 50 in* Seomae* mugwort (Table 6). Representative chromatograms of both mugwort species are shown in Supplemental Figure 1 in the Supplementary Material available online at http://dx.doi.org/10.1155/2015/916346. Intuitively, it is clear that* Seomae* mugwort should have more diverse profiles of volatiles given the numbers of compounds listed in the table and identified in chromatograms, as well as their peak areas. This was further supported by olfactometry analysis by three trained panels. Strong intensities of* Seomae* mugwort were recorded mostly between 12 min and 21 min of the aromagram (Supplemental Figure 1(C)). Notably, within this range of retention times, a few volatile chemicals present in* Seomae* mugwort had significantly higher peak areas. For instance, terpenic compounds (e.g., *α*-terpinolene and *α*-terpinene) were significantly more abundant in* Seomae* mugwort than in* A. princeps*; most of these compounds were not detected in* A. princeps* (e.g., *α*-terpinene, 1,8-cineole, camphor, and 4-terpineol; Table 6). It has been reported that these terpenic compounds possess characteristic woody, citrus, floral, and herbal flavors [19], which possibly confer more favorable sensory characteristics when consumed in the present study. Importantly, the sensory attributes of volatile compounds are difficult to predict due to potential associations between aromas of different compounds (e.g., synergistic or masking effects) [19]. In the olfactometry analysis, we only recorded the aroma intensities but were unable to assess their flavor descriptions and acceptability. Hence, comparative sensory evaluation of* Seomae* mugwort and* A. princeps* was performed.

**Figure 1:**
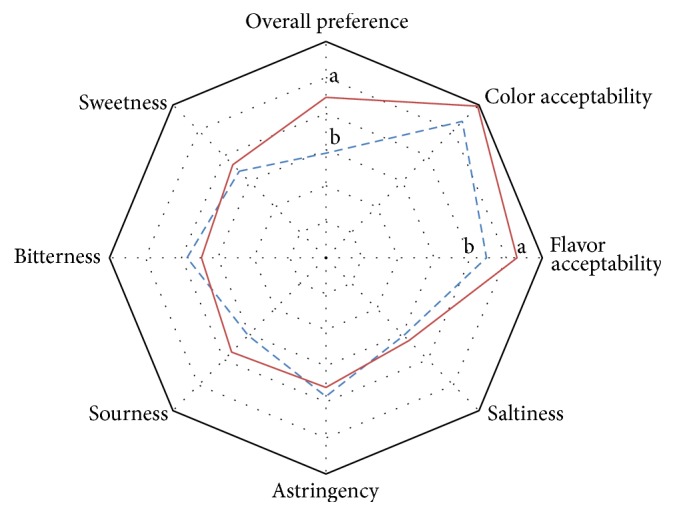
Comparison of the sensory profiles of mugwort tea prepared with either* Artemisia princeps* Pamp. or* Seomae* mugwort (a Korean native variety of* Artemisia argyi* H. Lév. & Vaniot). A total of 15 participants used LAM scales for perceived color acceptability, flavor acceptability, saltiness, bitterness, sourness, astringency, sweetness, and overall preference. Dashed line and solid line indicate* A. princeps* and* Seomae* mugwort, respectively. Preference scales ranged from 0 (greatest imaginable dislike) to 15 (greatest imaginable like). Different superscript letters indicate statistical significance of the differences between* Seomae* mugwort and* A. princeps* groups, tested by Student's *t*-test using the SAS. *P* values less than 0.05 were considered statistically significant.

To examine the potential for the practical use of* mugwort tea* as a* nutritious drink, we prepared tea samples from both mugwort species.* As mentioned above, the participants were asked to evaluate perceived preference for each mugwort tea. Subjects evaluated color acceptability prior to consuming the samples. Then, other perceived qualities (flavor acceptability, saltiness, bitterness, sourness, astringency, sweetness, and overall preference) were evaluated after sample consumption by using the LAM scales of 0–15 points. We did not find any differences in sweetness (6.8 ± 1.8 and 7.3 ± 1.2 for* A. princeps* and* Seomae* mugwort), bitterness (7.7 ± 1.5 and 6.9 ± 2.1 for* A. princeps* and* Seomae* mugwort), sourness (6.1 ± 2.2 and 7.4 ± 2.4 for* A. princeps* and* Seomae* mugwort), astringency (7.7 ± 2.1 and 7.2 ± 2.2 for* A. princeps* and* Seomae* mugwort), and saltiness (6.1 ± 2.4 and 6.5 ± 1.8 for* A. princeps* and* Seomae* mugwort) between samples. There were, however, significant differences in overall preference (5.8 ± 0.9 and 8.9 ± 1.1 for* A. princeps* and* Seomae* mugwort; *P* < 0.05) and flavor acceptability (8.9 ± 1.1 and 10.6 ± 1.0 for* A. princeps* and* Seomae* mugwort; *P* < 0.05, Figure 1), which may be due to the differences in the profiles of volatile compounds between* Seomae* mugwort and* A. princeps*, in particular the difference in terpenic compounds (Table 6). Of many properties, we were specifically interested in “bitter taste” and “astringency,” which may impact consumers' preference and palatability. In the analysis of free amino acids, we found that the content of branched-chain amino acids was slightly higher in* Seomae* mugwort than in* A. princeps* (259.20 mg/100 g versus 223.53 mg/100 g; Table 2). Branched-chain amino acids (leucine, isoleucine, and valine) are known to confer bitter taste [20]. However, our results indicate that there was no difference in such unfavorable tastes between these mugwort species. No significant correlation was found between tested sensory attributes and the frequency of tea consumption as well as participants' sex (data not shown). Considering the small number of participants, further investigations may be warranted to clarify and confirm the observed trends. Furthermore, it would be interesting to include another type of tea (e.g., green tea) in sensory evaluation as a control for a direct comparison with its sensory attributes. Lastly, given the nature of sensory evaluation, it is also possible that perceived attributes relatively vary with individuals; thus, descriptive sensory evaluation with trained panelists might be warranted in the future.

## 4. Conclusions

In the present study, we compared the nutritional characteristics and sensory attributes of* Seomae* mugwort, a native mugwort variety of* A. argyi* cultivated in Namhae County in South Korea, and those of* A. princeps*. The native variety showed (1) higher contents of essential amino acids without compromising flavor, (2) higher amount of polyunsaturated fatty acids, likely due to an increased content of C18:3 *ω*-3, (3) better radical scavenging activity against DPPH and higher vitamin C/total phenolic compound contents, and (4) more diverse volatile compounds with more favorable sensory attributes when consumed as tea. Given that scant information is available regarding the* Seomae* mugwort and its biological, chemical, and sensory characteristics, the results of this study may provide important preliminary data for further industrial and research applications of this mugwort variety.

## Supplementary Material

Representative total ion chromatograms of volatile compounds and aromagram of *Seomae* mugwort.

## Figures and Tables

**Table 1:** Demographic information of study participants and frequency of tea consumption^(a)^.

	Percentage (*n*)
Gender	
Male	33 (5)
Female	67 (10)

Age	
19–29	87 (13)
30–40	13 (2)
≤40	0

Frequency of tea consumption (per month)	
Never	13 (2)
≥5 times	60 (9)
≥10 times	27 (4)
≥20 times	0 (0)
Daily	0 (0)

^(a)^A total of 15 participants were recruited from the Gyeongnam National University of Science and Technology through fliers. The protocol was approved by the University Institutional Review Board and written consent forms were obtained from the participants in advance ofcollecting data.

**Table 2:** Free amino acids profile of *Artemisia princeps* Pamp. and *Seomae* mugwort^(a)^.

	*A. princeps *	*Seomae* mugwort
Essential amino acid (mg/100 g of dried material)		
Histidine	7.18 ± 0.16^a^	2.54 ± 0.06^b^
Phenylalanine	66.05 ± 0.26^b^	93.78 ± 0.74^a^
Valine	102.71 ± 1.97^b^	167.07 ± 0.85^a^
Leucine	59.26 ± 0.65^a^	44.51 ± 0.60^b^
Isoleucine	61.56 ± 0.89^a^	47.62 ± 00.57^b^
Threonine	22.20 ± 1.03^a^	15.12 ± 0.29^b^

Nonessential amino acid (mg/100 g of dried material)		
Arginine	29.47 ± 0.68^a^	20.55 ± 0.32^b^
*γ*-Aminobutyric acid	12.60 ± 0.18^b^	48.52 ± 0.87^a^
Alanine	86.90 ± 0.88^a^	34.29 ± 0.60^b^
Cysteine	4.42 ± 0.30	4.52 ± 0.25
Glutamic acid	23.59 ± 0.68^b^	33.45 ± 0.36^a^
Tyrosine	7.62 ± 0.13^b^	10.77 ± 0.38^a^
Glycine	4.57 ± 0.25^b^	11.39 ± 0.35^a^
*β*-Alanine	16.18 ± 0.78	15.96 ± 0.09
*α*-Aminobutyric acid	2.53 ± 0.44^b^	6.54 ± 0.37^a^
Aspartic acid	7.01 ± 0.51^b^	8.68 ± 0.29^a^
Serine	47.52 ± 0.61	47.48 ± 0.65

Total essential amino acid	318.93 ± 1.22^b^	370.64 ± 0.27^a^
Total nonessential amino acid	242.35 ± 2.24	242.15 ± 2.80
Total free amino acid	561.28 ± 3.30^b^	612.79 ± 2.97^a^

^(a)^Data represents the mean ± SD (*n* = 3). Different superscript letters indicate statistical significance of the differences between *Seomae* mugwort and *A. princeps* groups, tested by Student's *t*-test using the SAS. *P* values less than 0.05 were considered statistically significant.

**Table 3:** Method validation of fatty acids analysis: % accepted values and % relative standard deviations (RSD) determined using SRM 1849a.

Fatty acids	% weight			% of accepted value^(d)^	% RSD^(e)^
	Accepted value^(a)^	Analytical value^(b)^	Bias^(c)^		
C14:0	4.76 ± 0.14	4.79 ± 0.13	−0.03	100.63	2.71
C16:0	9.89 ± 1.10	9.81 ± 0.21	0.08	99.19	2.14
C16:1 *ω*-7	0.12 ± 0.01	0.13 ± 0.01	−0.01	108.33	7.69
C18:0	4.21 ± 0.10	4.25 ± 0.05	−0.04	100.95	1.18
C18:1 *ω*-9	50.37 ± 5.51	50.45 ± 2.72	−0.08	100.16	5.39
C18:1 *ω*-7	1.02 ± 0.03	1.03 ± 0.05	−0.01	100.98	4.85
C18:2 *ω*-6	25.95 ± 2.11	25.82 ± 1.10	0.13	99.50	4.26
C18:3 *ω*-3	0.42 ± 0.01	0.46 ± 0.02	−0.04	109.52	4.35
C20:0	0.24 ± 0.03	0.26 ± 0.01	−0.02	108.33	3.85
C20:1 *ω*-9	2.51 ± 0.26	2.52 ± 0.05	−0.01	100.40	1.98
C22:0	0.34 ± 0.01	0.32 ± 0.01	0.02	94.12	3.13
C24:0	0.17 ± 0.01	0.16 ± 0.01	0.01	94.12	6.25

^(a)^The accepted value was calculated using the certified fatty acids content of SRM 1849a based on % weight.

^(b)^Data represents the mean ± SD (*n* = 3). ^(c)^Bias = accepted value − analytical value. ^(d)^The ratio of the analytical value to accepted value expressed as a percentage. ^(e)^RSD indicates interday relative standard deviation (SD × 100/mean) of analytical values.

**Table 4:** Comparison of fatty acid profiles between *Artemisia princeps* Pamp. and *Seomae* mugwort^(a)^.

Fatty acids	*A. princeps *	*Seomae* mugwort
C16:0	8.73 ± 0.06^b^	18.82 ± 0.15^a^
C16:1	0.23 ± 0.01^b^	2.04 ± 0.05^a^
C18:0	3.54 ± 0.04^a^	1.66 ± 0.07^b^
C18:1	34.91 ± 0.06^a^	5.09 ± 0.09^b^
C18:2	27.56 ± 0.07^a^	15.73 ± 0.12^b^
C20:0	2.53 ± 0.04^b^	3.63 ± 0.13^a^
C18:3 *ω*-6	9.83 ± 0.06^b^	36.36 ± 0.20^a^
C22:0	8.58 ± 0.14^b^	10.91 ± 0.09^a^
C24:0	4.08 ± 0.14^b^	5.76 ± 0.07^a^

SFA^(b)^	27.47 ± 0.08^b^	40.79 ± 0.10^a^
MUFA^(c)^	35.14 ± 0.03^a^	7.12 ± 0.07^b^
PUFA^(d)^	37.39 ± 0.06^b^	52.09 ± 0.16^a^

^(a)^Data represents the mean ± SD (*n* = 3). Different superscript letters indicate statistical significance of the differences between *Seomae* mugwort and *A. princeps* groups, tested by Student's *t*-test using the SAS. *P* values less than 0.05 were considered statistically significant. ^(b)^SFA: saturated fatty acids. ^(c)^MUFA: monounsaturated fatty acids. ^(d)^PUFA: polyunsaturated fatty acids.

**Table 5:** Total phenolic contents, vitamin C contents, and antioxidative capacities of *Artemisia princeps* Pamp. and *Seomae* mugwort^(a)^.

	*A. princeps *	*Seomae* mugwort
Total phenolic content (mg/100 g of dried sample)^(b)^	49.12 ± 1.23^b^	74.53 ± 2.08^a^
IC_50 _in DPPH radical scavenging (mg)^(c)^	0.82 ± 0.12^a^	0.55 ± 0.09^b^
Vitamin C content (mg/100 g of dried sample)^(d)^	100.6 ± 2.2^b^	209.1 ± 3.2^a^

^(a)^Data represents the mean ± SD (*n* = 3). Different superscript letters indicate statistical significance of the differences between *Seomae* mugwort and *A. princeps* groups, tested by Student's *t*-test using the SAS. *P* values less than 0.05 were considered statistically significant. ^(b)^The total phenolic contents of samples were measured using Folin-Ciocalteu's reagent as described in the Materials and Methods. ^(c)^The IC_50_ values of *A. princeps* and *Seomae* mugwort were calculated and compared using a typical 2,2-diphenyl-1-picrylhydrazyl (DPPH) assay. ^(d)^The vitamin C was analyzed using the HPLC as described in the Materials and Methods.

**Table 6:** Volatile compounds present in *Artemisia princeps* Pamp. and *Seomae* mugwort^(a)^.

Peak number^(b)^	Compounds^(c)^	Retention time (min)	Peak area ×10^3^	
			*A. princeps *	*Seomae* mugwort
1	Propanoic acid methyl ester	3.32	4,655.5 ± 502.1^b^	43,759.7 ± 1,202.3^a^
2	Acetic acid ethyl ester	3.91	873.6 ± 90.2^b^	8,365.1 ± 902.1^a^
3	2,3-Dimethyl pentane	4.79	89.1 ± 84.3^b^	984.5 ± 42.5^a^
4	Butyl ethyl ether	5.39	478.3 ± 63.1^b^	4,321.9 ± 472.5^a^
5	Diethyl sulfide	5.51	61.5 ± 33.2^b^	1,687.2 ± 202.9^a^
6	Acetal	6.29	1,122.9 ± 172.6^b^	13,265.2 ± 1,502.5^a^
7	2-Methyl-2-hexanol	6.98	Not detected^b^	1,178.4 ± 227.3^a^
8	Valeric acid methylbutyl ester	7.07	1,646.2 ± 216.2^b^	19,573.6 ± 1,312.4^a^
9	Methylbenzene	7.32	372.7 ± 39.3^b^	3,455.6 ± 482.1^a^
10	2-Furancarboxaldehyde	9.45	53.3 ± 32.1^b^	953.3 ± 113.2^a^
11	Chlorobenzene	9.87	59.5 ± 29.6^b^	757.16 ± 221.5^a^
12	2-Hexenal	10.13	Not detected^b^	1,323.3 ± 160.5^a^
13	Ethyl benzene	10.40	1,253.9 ± 264.3^b^	15,135.1 ± 1,302.1^a^
14	*m*-Xylol	10.67	42.9 ± 40.7^b^	624.01 ± 129.4^a^
15	*o*-Xylol	11.47	89.3 ± 66.2^b^	1,628.5 ± 278.4^a^
16	*α*-Terpinolene	12.62	Not detected^b^	60,468.9 ± 2,532.8^a^
17	*α*-Pinene	12.85	252.9 ± 102.5^b^	5,512.4 ± 762.0^a^
18	Camphene	13.35	Not detected^b^	2,842.3 ± 388.7^a^
19	Sabinene	14.18	49.8 ± 36.1^b^	357.3 ± 94.4^a^
20	*β*-Pinene	14.29	220.3 ± 100.5^b^	1,473.5 ± 233.5^a^
21	1-Octen-3-ol	14.34	Not detected	3,433.3 ± 582.3^a^
22	*β*-Myrcene	14.72	119.4 ± 84.5^a^	Not detected^b^
23	Yomogi alcohol	15.08	Not detected^b^	288,651.3 ± 1,321.1^a^
24	*α*-Terpinene	15.57	Not detected^b^	1,863.8 ± 282.4^a^
25	*o*-Cymene	15.83	Not detected^b^	1,483.3 ± 248.5^a^
26	D-Limonene	15.96	75.9 ± 63.1^b^	750.3 ± 121.5^a^
27	1,8-Cineole	16.05	Not detected^b^	32,351.2 ± 1,321.8^a^
28	2,4-Hexadiene	16.17	Not detected^b^	5,933.3 ± 567.3^a^
29	Phenyloxirane	16.42	695.1 ± 111.8^a^	Not detected^b^
30	Benzeneacetaldehyde	16.43	Not detected^b^	5,493.2 ± 484.3^a^
31	*γ*-Terpinene	16.90	Not detected^b^	1,384.6 ± 233.2^a^
32	*cis*-*β*-Terpineol	17.21	Not detected^b^	617.9 ± 171.3^a^
33	Artemisia alcohol	17.78	Not detected^b^	533,734.3 ± 8,242.0^a^
34	*β*-Linalool	18.15	136.7 ± 70.3^b^	17,562.3 ± 1,382.4^a^
35	Nonanal	18.25	80.7 ± 29.5^a^	Not detected^b^
36	Camphor	19.57	Not detected^b^	4,463.87 ± 529.4^a^
37	4-Terpineol	20.49	Not detected^b^	4,215.52 ± 498.5^a^
38	*β*-Fenchyl alcohol	20.88	Not detected^b^	2,583.98 ± 200.4^a^
39	Indole	23.69	144.0 ± 101.1^b^	1,073.78 ± 218.3^a^
40	*δ*-Elemene	24.87	75.4 ± 43.1^a^	Not detected^b^
41	Eugenol	25.34	Not detected^b^	13,037.30 ± 1,009.3^a^
42	*α*-Copaene	25.90	325.0 ± 112.2^b^	2,643.10 ± 183.6^a^
43	*β*-Bourbone	26.16	65.25 ± 45.6^b^	2,933.01 ± 438.3^a^
44	*β*-Elemene	26.27	845.4 ± 205.1^a^	Not detected^b^
45	Caryophyllene	27.06	13,728.3 ± 1,225.3^b^	85,473.18 ± 5,384.5^a^
46	*β*-Copaene	27.26	Not detected^b^	1,417.52 ± 135.8^a^
47	*α*-Amorphene	27.65	60.5 ± 60.9^a^	Not detected^b^
48	*cis*-*β*-Farnesene	27.75	1,890.6 ± 210.9^b^	2,483.2 ± 499.3^a^
49	*α*-Humulene	27.88	3,921.7 ± 673.3^b^	9,065.3 ± 886.1^a^
50	*γ*-Muurolene	28.37	Not detected^b^	1,646.7 ± 245.3^a^
51	*γ*-Curcumene	28.38	998.1 ± 89.0^a^	Not detected^b^
52	*β*-Cubebene	28.54	16,826.3 ± 1,533.2^b^	29,434.57 ± 5,553.7^a^
53	*β*-Selinene	28.68	Not detected^b^	8,386.7 ± 1,334.3^a^
54	Zingiberene	28.75	6,225.4 ± 562.1^a^	Not detected^b^
55	Germacrene B	28.91	1,347.8 ± 113.2^a^	Not detected^b^
56	*α*-Farnesene	28.99	889.5 ± 82.0^a^	Not detected^b^
57	*β*-Bisabolene	29.07	200.2 ± 121.2^a^	Not detected^b^
58	*γ*-Cadinene	29.29	353.5 ± 178.1^b^	3,976.39 ± 529.9^a^
59	*δ*-Cadinene	29.47	1,345.4 ± 203.1^b^	4,073.7 ± 587.9^a^
60	*α*-Cadinene	29.82	211.4 ± 52.2^a^	Not detected^b^
61	*trans*-*β*-Farnesene	30.33	158.2 ± 78.0^a^	Not detected^b^
62	Nerolidol	30.34	Not detected^b^	3,122.5 ± 443.9^a^
63	Caryophyllene oxide	30.95	315.3 ± 192.1^a^	Not detected^b^
64	Diethyl phthalate	31.02	233.6 ± 54.2^a^	Not detected^b^
65	*α*-Guaiene	31.30	Not detected^b^	1,347.3 ± 309.4^a^
66	tau-Muurolol	32.20	349.1 ± 120.2^a^	Not detected^b^

^(a)^Data represents the mean ± SD (*n* = 3). Different superscript letters indicate statistical significance of the differences between *Seomae* mugwort and *A. princeps* groups, tested by Student's *t*-test using the SAS. *P* values less than 0.05 were considered statistically significant. ^(b)^Peak numbering was determined by the order of elution. ^(c)^The gas chromatographic retention data and mass spectral data were compared to those of authentic samples and library compounds, respectively.
