# Supplementary material for: Characterization of Nutritional Composition, Antioxidative Capacity, and Sensory Attributes of Seomae Mugwort, a Native Korean Variety of Artemisia argyi H. Lév. & Vaniot
Source: J Anal Methods Chem. 2015 Oct 13;2015:916346. doi: 10.1155/2015/916346 (PMC4621344; doi:10.1155/2015/916346)
Supplement: Supplementary file 1 — Representative total ion chromatograms of volatile compounds and aromagram of Seomae mugwort. [file 916346.f1.pdf]

Supplemental Figure 1.

Representative total ion chromatograms of volatile compounds present in *Artemisia princeps* Pamp. (A), *Seomae* mugwort (B), and aromagram of *Seomae* mugwort (C)

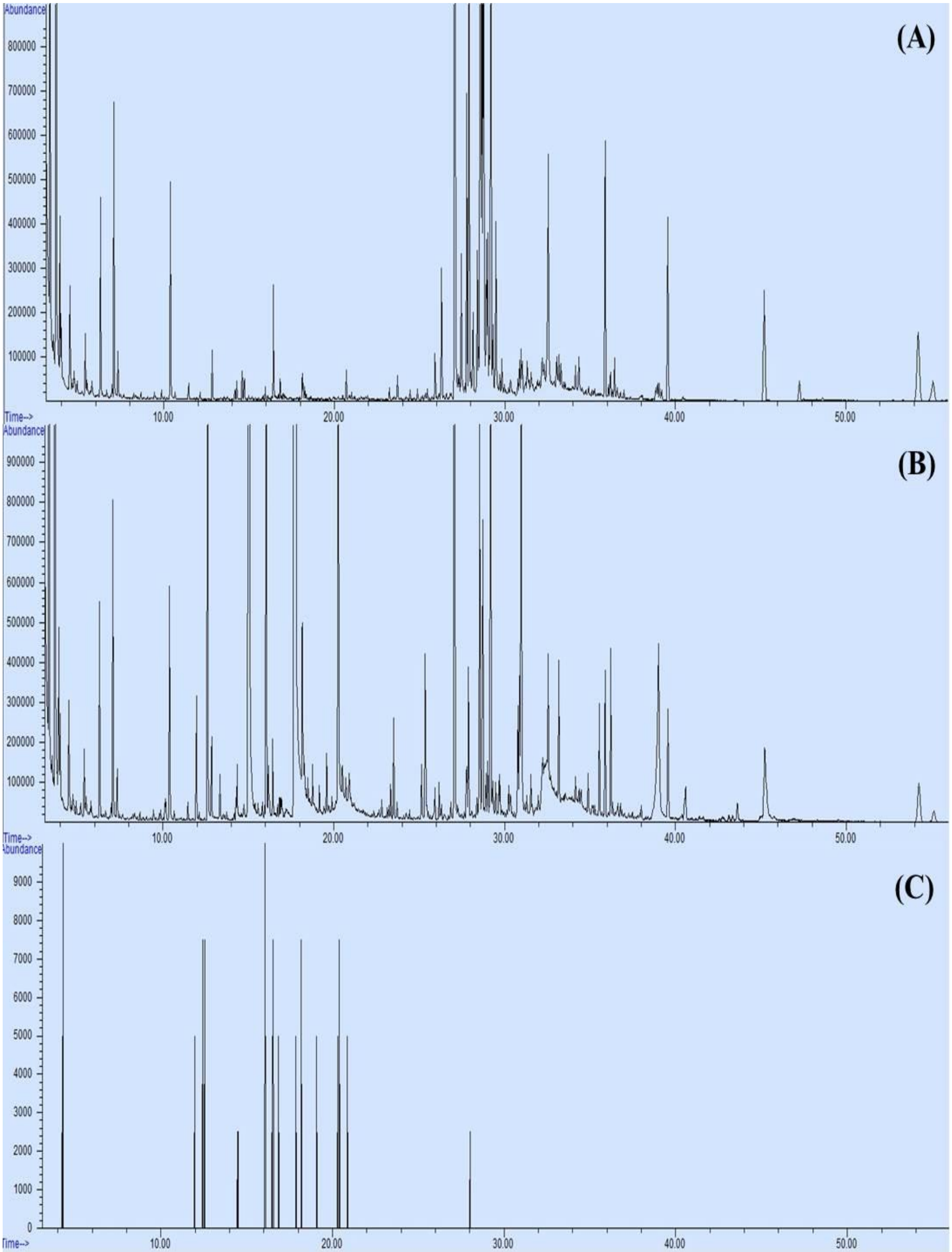

Supplemental Figure 1. Kim JK *et al.*
